# Social Determinants of Health in EMS Records: A Mixed-methods Analysis Using Natural Language Processing and Qualitative Content Analysis

**DOI:** 10.5811/westjem.59070

**Published:** 2023-08-08

**Authors:** Susan J. Burnett, Rachel Stemerman, Johanna C. Innes, Maria C. Kaisler, Remle P. Crowe, Brian M. Clemency

**Affiliations:** * Jacobs School of Medicine and Biomedical Sciences, University at Buffalo, The State University of New York, Department of Emergency Medicine, Buffalo, New York; † ESO, Austin, Texas

## Abstract

**Introduction:**

Social determinants of health (SDoH) are known to impact the health and well-being of patients. However, information regarding them is not always collected in healthcare interactions, and healthcare professionals are not always well-trained or equipped to address them. Emergency medical services (EMS) professionals are uniquely positioned to observe and attend to SDoH because of their presence in patients’ environments; however, the transmission of that information may be lost during transitions of care. Documentation of SDoH in EMS records may be helpful in identifying and addressing patients’ insecurities and improving their health outcomes. Our objective in this study was to determine the presence of SDoH information in adult EMS records and understand how such information is referenced, appraised, and linked to other determinants by EMS personnel.

**Methods:**

Using EMS records for adult patients in the 2019 ESO Data Collaborative public-use research dataset using a natural language processing (NLP) algorithm, we identified free-text narratives containing documentation of at least one SDoH from categories associated with food, housing, employment, insurance, financial, and social support insecurities. From the NLP corpus, we randomly selected 100 records from each of the SDoH categories for qualitative content analysis using grounded theory.

**Results:**

Of the 5,665,229 records analyzed by the NLP algorithm, 175,378 (3.1%) were identified as containing at least one reference to SDoH. References to those SDoH were centered around the social topics of accessibility, mental health, physical health, and substance use. There were infrequent explicit references to other SDoH in the EMS records, but some relationships between categories could be inferred from contexts. Appraisals of patients’ employment, food, and housing insecurities were mostly negative. Narratives including social support and financial insecurities were less negatively appraised, while those regarding insurance insecurities were mostly neutral and related to EMS operations and procedures.

**Conclusion:**

The social determinants of health are infrequently documented in EMS records. When they are included, they are infrequently explicitly linked to other SDoH categories and are often negatively appraised by EMS professionals. Given their unique position to observe and share patients’ SDoH information, EMS professionals should be trained to understand, document, and address SDoH in their practice.

Population Health Research Capsule What do we already know about this issue?

*The ability of emergency medical services (EMS) personnel to assess patients’ social determinants of health (SDoH) can have a great impact on patient care.*

What was the research question?

*We sought to evaluate the presence, appraisal, and relationships of SDoH documentation in EMS records.*

What was the major finding of the study?

*Of 5,665,229 adult patient EMS records we analyzed, 3.1% were identified as containing at least one reference to SDoH.*

How does this improve population health?

*Understanding how EMS personnel recognize and document patients’ SDoH is key to identifying their diverse needs and expanding out-of-hospital care options.*

## INTRODUCTION

Social determinants of health (SDoH), including housing, employment, education, income, neighborhoods, access to healthcare, and education are known to impact the health and well-being of patients, yet these variables are not always accounted for during interactions with the healthcare system.^1^^,^^2^ While unmet basic social needs have measurable impacts on individuals, communities, and the public health system,^1^^,^^3^^,^^4^ little guidance exists for healthcare clinicians to address patients’ SDoH, and current strategies focus on the population and policy levels.^5^ Greater attention is needed to individual-level social issues, as leaving them unaddressed leads to poor clinical outcomes, health disparities, and increased healthcare costs.^3^ Patients with unmet social needs frequently access healthcare through hospitals and, particularly, emergency departments (ED) only, warranting an increased focus on SDoH in emergency medicine (EM).^6^^–^^8^

Emergency medical services (EMS) professionals operating at the intersection of public safety, public health, and healthcare are uniquely positioned to observe and attend to SDoH in productive and influential ways.^9^ They use their perceptions of patients’ social and physical environments to aid in medical decision-making, such as determining whether to transport the patient to the hospital or not.^10^ Additionally, conveying information to ED staff about factors such as unsafe housing, lack of access to medications, or inability of patients to safely care for themselves can ultimately affect whether or not that patient can be safely discharged from the ED. Nevertheless, information exchange through verbal hand-offs from EMS professionals to ED nursing staff and subsequent reporting to additional hospital personnel often results in lost information and miscommunication.^11^^–^^19^

Electronic health record documentation by EMS is a reliable channel through which information about a patient’s SDoH can be shared with all healthcare personnel associated with the patient’s hospitalization.^20^ There is a paucity of SDoH information in EMS records for pediatric patients,^21^ but the presence, appraisal, and connections of SDoH information in EMS records for adult patients is unknown. Our objective in this study was to determine the presence and frequency of SDoH documentation in adult EMS records and understand the ways in which those insertions are referenced, appraised, and linked to other determinants.

## METHODS

### Study Design and Setting

We retrospectively analyzed 9-1-1 records for adult patients (≥18 years) in the 2019 ESO Data Collaborative public-use research dataset. The ESO Data Collaborative consists of de-identified prehospital electronic patient care records created by EMS personnel during the course of patient care. All EMS agencies that contribute to the dataset agree to share their de-identified data for research and benchmarking. Annual research datasets are made available free of charge following a proposal process and review by an institutional review board (IRB). We selected this database for the diversity of included practice settings and the ability to request free-text narratives. In 2019, this database contained more than eight million records from 1,322 EMS agencies with encounters across all four US Census regions (South: 58%; Midwest: 22%; West: 16%; and Northeast: 5%) and 6% of encounters occurring in rural settings. A total of 31% of encounters occurred in communities classified within the most vulnerable quartile of socioeconomic status based on the US Centers for Disease Control and Prevention’s social vulnerability index.^22^ This study was designated not human subjects research by the State University of New York at Buffalo IRB.

### Selection of Cases

All cases, regardless of complaint or disposition, were screened. We used a multilabel classification machine-learning model which, when identifying SDoH topics, has area under the curve receiver operating characteristics of 93.9.^23^ This natural language processing (NLP) algorithm has a framework of applications to EM and EMS records.^21^^,^^23^ By applying this algorithm, we identified free-text narratives containing documentation of at least one SDoH from categories associated with food, housing, employment, insurance, financial, and social support insecurities.

### Measures and Analysis

We performed descriptive statistical analysis and randomization of records for qualitative analysis using Stata MP version 17.0 (StataCorp LLC, College Station, TX). From the corpus produced by the NLP algorithm, a random sample of 100 narratives was chosen from each determinant category for qualitative content analysis. Using an interpretive paradigm,^24^ the three study team members read each narrative to understand the first-person perspectives of the EMS professionals who documented their interactions with patients in the records they kept. This approach was necessary to facilitate a hermeneutical approach and understand the social construction of those EMS professionals’ experiences on the calls about which they documented. Team members used a grounded theory framework^24^^–^^26^ to describe the content found in the EMS records.

Three researchers (JCI, an ED attending, EMS physician, and paramedic; MCK, an ED attending and EMS fellow; and SJB, a paramedic and EMS educator) reviewed narratives, performed primary coding to understand the content of the EMS records, and further immersed themselves in the data by discussing their findings for each category with the other qualitative-analysis study team members.^24^^,^^27^ During this data immersion phase, codes were developed. For example, patients who were documented as not having eaten in several days were coded as food insecure (if their cases were not otherwise categorized in the food insecurity determinant category by the NLP corpus), or patients who reported they could not afford medications were coded as financially constrained. Then, using the constant comparative method,^24^^,^^28^ researchers organized the primary codes into secondary codes to synthesize social topics illustrated by the data throughout the determinant categories. For example, patients who reported financial constraints were categorized as having accessibility problems. Researchers further collaborated to assure that the content of the EMS records was represented by the social topics and each concept was robustly supported by data.^29^

In a separate round of purposive coding, researchers looked for documentation of other insecurities in each determinant category to determine the relationship, frequency, and directionality of their reference. For example, if a narrative was determined by the NLP corpus to contain information about social support insecurities, but the patient was documented as not having eaten in several days, they were coded as food insecure, as referred to in the social support category. Additionally, the team members qualitatively appraised each narrative to identify the valence of the narratives as a means of further understanding the EMS professionals’ perspectives. To limit potential perceptions of bias, the qualitative researchers frequently checked in with themselves and the others as a means of reflexivity.^24^^,^^28^ They verified that their interpretations and models were representative of the data and that their own and the others’ experiences and biases did not result in misinterpretation. They also collaboratively built models and interrogated their data to assure their findings were strongly supported by the data from the EMS record narratives.

## RESULTS

Of 5,665,229 records analyzed by the NLP algorithm, 175,378 (3.1%) were identified as containing at least one reference to a SDoH. Of the records in this corpus, 171,740 (97.93%) contained only one identifiable reference to SDoH, while 3,580 records (2.04%) contained two identifiable references; 57 (0.03%) contained three identifiable references; and one of the records (<0.01%) contained four identifiable references to SDoH. Records containing appearances of SDoH in the corpus were as follows: housing (52.28%); employment (33.06%); general financial (8.04%); insurance (4.73%); social support (3.86%); and food (0.14%).

### Social Topics Illustrated in Emergency Medical Services Records

Similar SDoH topics were identified throughout the various social determinant categories. These SDoH topics were accessibility, mental health concerns, physical health concerns, and substance use. Examples of the social topics’ appearances in each determinant category can be found in the Table.

**Table 1.** Representative quotes for each social topic within each determinant category.

		Social Topics			
		Accessibility	Mental health	Physical health	Substance use
SDoH categories
	Food	“She had been standing in line outside <the food bank> for approx. 30min, felt light headed <sic>, and passed out… states she hasn’t eaten since yesterday.”	“<The patient> advised that he was suicidal because his roommates had been bullying him by taking his belongings, including his phone, food stamps, etc. <He> also advised that he was feeling homicidal, but that those feelings had passed.”	“<The patient> states she vomited yesterday and her chest hurts today… she went to WIC today to get food at the food bank.”	“He relays he was on a meth binge over the weekend and feels tired but needs to be here to get his food stamps for the week.”
	Employment/ income	Male at day labor staffing building “was standing next to the counter and began having a seizure… <the patient> states he does have a hx of seizures and has been off his Keppra for quite some time now, ‘states he can’t afford it.’”	Patient living in a community facility “started having hallucinations… <The patient> was talking about baby geese that staff did not see.”	“<The patient> states he felt dizzy and went to ground from seated position. <The patient> states he feels fine and is refusing all treatment or transport… <he> states he is a diabetic but <cannot> afford medical bill<s>. <His mother> states she will take him to the ED.”	Dispatched to a motel for a stroke: “<the patient> is homeless but came to stay with her sister… <the patient> admitted to smoking marijuana this morning… She advised <of medical history including> drug use and she hasn’t drank <sic> alcohol in several days… or eaten in a few days.”
	Financial	The patient presented as “hyperglycemic, hypertensive, <with altered mental status>… He has a history of hypertension and diabetes, but it is not controlled with medications, as he has been unable to afford them for several months.”	A representative from the patient’s bank called because the patient “had made suicidal comments to them during a phone conversation involving a bill… <the patient> states that she is depressed about the passing of her husband and has been having financial issues as well.”	“The patient stated she has a history of COPD and had complications <with> breathing for the past 12 hours… The patient stated she is normally on home oxygen but has been unable to afford her medications this month.”	After receiving naloxone, “she began to come around. She admitted to alcohol use but no other drugs… she wanted to sign out of the hospital before she accumulated any bills because she felt they could not afford them.”
	Insurance	Patient “reporting a headache that started last night at 1900 hours due to withdrawals from Depakote and amitriptyline. She reports she has been out approx. one week due to the cost because she lost her Medicaid.”	Call for psychiatric complaints and crew found the patient requesting assistance “getting her Medicaid … pt was being uncooperative and yelling angrily… pt refused <transport>, becoming angry again and stating she didn’t want to go to the ED and that she just wanted her Medicaid again.”	After an MVC, a patient complained of “lower back pain… Patient stated she wanted to be check out by EMS but refuse to be transport by EMS due to no insurance.”	The patient left an ED for a heroin overdose … “patient became belligerent and aggressive when asked about drug use since leaving <hospital> patient stated the blood <on his face> was from him vomiting blood and the abrasions were for <an> allergic reaction to the <heroin> … patient does not have insurance.”
	Social support	Patient at a physician’s office was found hypertensive. “The patient’s niece related that there has been a problem with the patient’s pharmacy and <the medications have not been in stock>.” The niece also reported the patient lives alone and, while she checks on him, “she feels that he is not taking them because he doesn’t remember to <take his medications>.”	The patient’s friend reported, “she started hiding in different places in the house… <then went> into a neighbor’s garage <to> hide from her friend …” When a police officer arrived, the patient “stated she did not want to be shot and put her hands over her head as if she was told to raise her hands.” After speaking with a mental health professional on scene, “a family friend came and … stated that she would take her to the hospital and pick up her medication.”	The patient called 911 for chest pain and reported inability “to sleep … nausea and felt like she might faint. She waited to see if she would improve. as time went by, she states she became more worried and finally decided to call 911.” She refused transport and “admits her anxiety was a significant factor in her calling 911. Pt frequently calls 911. Pt lives alone.” Her visiting nurse was due soon and the “patient does not believe her nurse will make her go as she is feeling better.”	An elderly female who lived alone was checked on by a friend who found her stating she had “progressively been feeling weaker over the past week … pt is normally able to walk and take care of herself with no assistance.” The patient’s friend also revealed the patient “is an alcoholic and was drinking heavily up until one week ago.”
	Housing	Call for patient at facility who seized: “staff states <the patient was without> his medications x1 week. <The patient> states they were stolen from him while he as at the homeless center.”	Homeless shelter staff called because the patient “was walking around with broken glass bottles and was paranoid.” The patient was “hyper paranoid. And stated, ‘they are trying to kill me.’ <The patient> advised that ‘some put a hit’ out on her and that someone has been following her.”	“Found a <female patient> at local women’s shelter with c/c of contractions, abdominal pain. <The patient> states she is 34 w pregnant with twins.”	“Patient advised he is addicted to meth and wants to get help with detox.” Upon arriving the homeless shelter, “he did about ½ gram of meth about two hours ago… he attempted to spray ‘bleach bathroom cleaner’ in his mouth to assist him in passing a drug test.”

*Approx.,* approximately; min, minute; *WIC,* Women, Infants and Children; *hx,* history; *ED,* emergency department; *pt,* patient; *MVC,* motor vehicle collision; *EMS,* emergency medical services; *w*, weeks.

#### Accessibility

All determinant categories included documentation of concerns about patients’ lack of access to services or goods, often because of an inability to afford or physically get to their needed interventions. Patients with employment/income insecurities were documented as unable to afford safe housing, medications, or medical care. Attending EMS professionals linked these financial constraints to the calls for help, particularly when ambulances were used as a means of transportation or facilitators for additional, non-urgent care, such as access to medications, treatments, or social services. It was also noted that financial barriers to access compounded existing health conditions because patients could not afford necessary medications or follow-up care. When other patients with documented financial insecurities encountered EMS for non-chronic conditions (eg, motor vehicle collisions or falls), they refused treatment and transport by EMS because of reported inabilities to afford ambulance or hospital bills.

When documenting food insecurities, EMS personnel wrote that patients could not readily afford or access nutritious foodstuffs. In some cases, patients were reported as selling, skipping, or misusing medications to divert funds to their food budgets. Housing insecurity information was documented because of patients’ lack of safe or permanent housing, which may have presented problems upon discharge. Insurance insecurities were documented because of a lack of access to health insurance, sometimes secondary to lack of employment; however, more often, EMS personnel wrote about patients’ using the 9-1-1 system to express their desires to obtain insurance or care covered by insurance. These patients explicitly requested help in signing up for insurance or referrals to other sectors of the healthcare system, and in one case a person called 9-1-1 for transport to a facility to have an already prescribed medical procedure because their insurance would not otherwise cover it as an outpatient treatment. In the documentation of social support insecurities, patients lacked family members, friends, or healthcare personnel to aid in safely caring for their conditions, accessing food, or finding safe living environments. When strong social support was available, access to resources and assistance was clearly documented in this determinant category.

#### Mental Health Concerns

When mental health concerns were documented, regardless of determinant category, EMS professionals conveyed they were precipitated by the insecurities. Patients with employment and food insecurities expressed depression or suicidality while patients with housing insecurities primarily described anxiety. Those with documented financial and insurance insecurities also reported suicidality, but the EMS professionals explicitly linked the cause of financial constraints as ongoing expenses of chronic health conditions. Additionally, there were cases of documented elder or financial abuse perpetrated on or by patients who interacted with EMS. Those with social support insecurities were primarily anxious or nervous about living alone, but these issues often were not the reasons for why the calls were made for emergency services.

#### Physical Health Concerns

Patients with employment and housing insecurities were often found outside, exposed to the elements, and with complaints related to those conditions. For example, many complained of pain in their legs or backs that could have been attributed to their frequent walking or hot- or cold-related issues. Some of these cases were results of calls for other conditions, but EMS crews often found these patients were more interested in shelter than medical care at the hospital. Those with food insecurities complained of weakness, hypoglycemia, or near or completed syncope, particularly while standing in line, awaiting access to a food bank. Some reported being without food because of financial problems, but others reported they had no other way of accessing goods without community resources. In some cases, patients reported prioritizing accessing medications over food. Those with financial and insurance insecurities most often complained of manifestations of their chronic conditions and inability to treat them. In some cases, patients with both determinant types had acute health concerns and many had diagnoses, but they could not afford or otherwise access the prescribed treatments. Patients with social support insecurities most often complained of acute medical- or trauma-related issues and an inability to properly address them. These patients were most often elderly patients who complained of weakness and falls.

#### Substance Use

Patients with documented employment and housing insecurities were more often described to be using or withdrawing from various substances. Those with food insecurities were also described as engaging in heavy use of substances while their ability to access or not access food was documented. Documentation of alcohol use with food, financial, housing, insurance, and social support insecurities were rarely associated with patients’ complaints or conditions, but they were frequently written about as part of the scene-setting descriptions.

### Relationships Between Determinant Categories

Within the social determinants categories, EMS personnel sometimes documented links to other determinant categories. For example, when a chart was flagged because of a documented food insecurity, EMS personnel included information about concurrent housing, financial, or social support insecurities. (See Figure for articulated relationships between determinant categories.) Some of these relationships were unidirectional (eg, insurance insecurities were linked to social support insecurities, but social support insecurities were not linked to insurance insecurities), although there were frequent bidirectional referrals between determinants but for varying reasons. For example, patients with financial insecurities refused EMS treatment and transport because they were documented as reporting a lack of insurance, but patients with insurance insecurities requested EMS transportation because they could not afford alternative treatment or transport.

**Figure 1.**
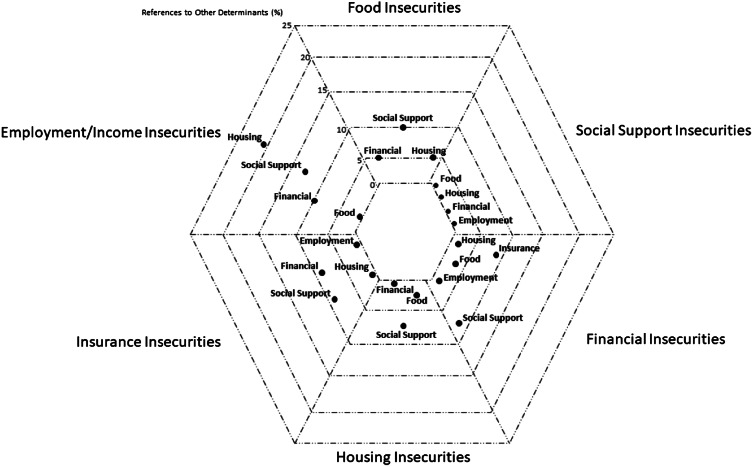
Explicit references to other determinant categories in charts (%).

Most often, however, the other determinants were not explicitly referenced in the documentation, but plausible explanations could be inferred from context. For example, in the case of a patient with housing insecurity, the EMS professional documented the patient had not eaten or taken their medications in several days. These elements could be indicative of food, insurance, or financial insecurities, but they were not explicitly cited.

### Appraisal of EMS Professionals’ Perspectives

When EMS professionals wrote about employment, food, and housing insecurities, most of the appraisals were negative and appeared to discursively position the patients as at fault for their 9-1-1 complaints and life conditions. Some of the content in these narratives was unnecessary and unrelated to the patients’ needs within the context of the EMS call. For example, in the case of a patient with housing insecurity who complained of lower extremity pain, the writer included several insertions about how the patient did not appear to have discomfort or unsteady gait and hypothesized the patient had ulterior motives for the call to 9-1-1. For those with insurance insecurity, most of the documentation in the EMS narratives included inability for patients or crew members to obtain the billing information for another part of the chart. Narratives for patients with financial or social support insecurities contained less perceivable negativity in the descriptions of those patients’ conditions.

Within the social support category, specifically, the narratives were generally much longer than in the other categories and the scenes were described with greater detail. Although some of the patients were documented as repeat customers, the overtone was positive, and the EMS professionals described their familiarity in association with the help they provided for the patients in the current and previous interactions. Only the social support insecurities category contained descriptions of the advocacy work provided by the EMS crews on the scene, including referrals to mobile integrated health or other community resources, assisting in patients’ errands, calling a patient’s primary healthcare physician for them, or delivering food from their own station so the patient could eat a meal. This category also contained richer descriptions of safety concerns for patients who lived in homes, generally alone, yet the reflection of safety concerns did not come up for patients who did not have homes, food, medications, or money.

## DISCUSSION

Understanding how EMS professionals document SDoH within electronic health records is vital for improving emergency care, subsequent treatment, and outcomes for patients with insecurities. Most often, SDoH information was not included in EMS records and, when present, was neither holistic nor interconnected to other insecurities that may impact a patient’s health and well-being. Additionally, EMS personnel often—likely unintentionally—wrote about patients’ insecurities with a negative valence or tone. Such negativity in health records has demonstrated downstream impressions of the patients themselves.^30^

Because EDs are venues that perform as safety nets for myriad health and social problems, there are proposals for employing social emergency care to screen and connect patients with the resources they need to address their insecurities.^6^^,^^31^ As members of the patients’ care teams, EMS professionals should be included in any efforts to collect and apply information about patients’ SDoH. Patient navigators and other hospital personnel collect data and add SDoH information to patients’ medical records,^31^^,^^32^ but hospital personnel should be aware of the EMS records’ content and the reduced likelihood of social desirability bias or influence by the EMS professionals.

Social EM is a burgeoning sub-field of EM^6^ and can be seen as a way for the EMS profession to expand its scope, as well. Calls to expand training and curricula for emergency practitioners^33^^,^^34^ can be extended to EMS professionals to improve the quantity and quality of SDoH-related content in EMS records. While addressing SDoH in all medical records, terminology should be consistent to avoid miscommunication.^8^ Systematic and prescribed formatting for verbal hand-offs from EMS to ED personnel have improved efficacy and information transmission,^11^^,^^16^^,^^18^^,^^35^ and the National Emergency Medical Services Information System (NEMSIS)^36^ has standardized and improved the collection of EMS data. The creation of a specific data collection tool can increase and improve information acquisition and neutral reporting of patients’ SDoH.

Additionally, recent initiatives to address patients’ needs and avoid unnecessary transports to overwhelmed EDs—such as the Emergency Triage, Treat, and Transport (ET3) Model^37^—by addressing SDoH through community engagement have resulted in fewer unnecessary calls to 9-1-1, fewer unnecessary visits to EDs, less out-of-service time for first responder units, and decreased incidence of patient falls.^38^^,^^39^ All EMS personnel should learn about the resources available in their own communities for addressing SDoH (eg, emergency shelters, food banks, or home healthcare and outreach organizations). If EMS professionals are aware of support services in their communities, they may be more likely to help their patients make necessary connections. Emergency medical services personnel of all levels and in all organization types should undergo specific training to recognize and address patients’ insecurities. Future research about EMS personnel’s knowledge about SDoH, their roles in this type of data collection, and perceptions of education about the topic may be informative to EMS and other fields’ implementation of interventions to improve longitudinal patient care.

Over the past several years, increased recognition and research linking emergency care and SDoH have significantly impacted the volume of literature in this salient area. These studies have examined SDoH and training,^33^^,^^34^^,^^40^^,^^41^ documentation and screening,^8^^,^^23^^,^^42^^–^^44^ interventions,^6^^,^^45^^,^^46^ homelessness,^47^ mental health,^48^^,^^49^ insurance types,^50^ and links to chronic or acute illness and injuries.^7^^,^^51^^–^^54^ Nearly all of these studies focused on EDs, which are not the only points of contact for all patients seeking emergency care and lack the perspective of seeing from where a patient hails. Emergency medical services personnel have the benefit of sharing in patients’ lived experiences and interacting with patients who may not subsequently present to EDs. This study provides a view from this novel vantage point.

## LIMITATIONS

The narratives analyzed in this study were from EMS records from various sources throughout the US, including first response agencies, transporting ambulance agencies, and flight EMS organizations. The certification levels of the EMS personnel were unknown during analysis. There may be differences in documentation of SDoH based on geographic location, organization types, or levels of training. Since this was a retrospective analysis of free-text narratives, we did not assess other sections of the records for information about patients’ SDoH. Given the de-identified nature of this dataset, it was also unclear whether individual agencies provided guidance to their personnel about SDoH or their documentation. Additionally, this analysis leveraged records from a large convenience sample of EMS agencies that use a single, privately owned electronic health record system; thus, the generalizability of these findings to communities served by EMS agencies using other documentation systems is unknown.

The use of three qualitative researchers and joint analysis sessions may have increased the potential for groupthink, which could have narrowed the focus on themes and concepts for the benefit of consensus-building. Future studies should evaluate whether these variables are associated with SDoH documentation and valence of EMS personnel’s perspectives. Use of the interpretive paradigm and hermeneutical approach could account for any potential differences in message reception and intention during documentation.

## CONCLUSION

Addressing social determinants of health can lead to improved health outcomes, reduced strain on healthcare systems, and decreased health spending. Emergency medical services professionals are uniquely positioned to collect and share information on patients’ SDoH through their documentation, but the overwhelming majority of EMS records lack such content. Education about SDoH and their relationships to one another, along with training on how to neutrally include such content in their documentation can be beneficial for EMS professionals and their patients. The creation of standardized educational content and documentation tools to collect SDoH information and collections of organizations’ community resources for addressing insecurities may improve EMS professionals’ awareness, documentation, and treatment of SDoH.
